# Evaluation of Glaucomatous Damage via Functional Magnetic Resonance Imaging, and Correlations Thereof with Anatomical and Psychophysical Ocular Findings

**DOI:** 10.1371/journal.pone.0126362

**Published:** 2015-05-13

**Authors:** Vanessa M. Gerente, Ruth R. Schor, Khallil T. Chaim, Marcelo de Maria Felix, Dora F. Ventura, Sergio H. Teixeira, Claudio L. Lottenberg, Edson Amaro, Augusto Paranhos

**Affiliations:** 1 Ophthalmology Department, Federal University of São Paulo, São Paulo, Brazil; 2 Hospital Israelita Albert Einstein, São Paulo, Brazil; 3 Experimental Psychology Department, Institute of Psychology, University of São Paulo, São Paulo, Brazil; Massachusetts Eye & Ear Infirmary, Harvard Medical School, UNITED STATES

## Abstract

**Purpose:**

To evaluate the functional magnetic resonance imaging (fMRI) response to binocular visual stimulation and the association thereof with structural ocular findings and psychophysical test results in patients with glaucoma, and controls.

**Methods:**

Cross-sectional study. Participants underwent a complete ophthalmic examination, including Humphrey 24-2 visual field (VF) testing and optical coherence tomography. Binocular VF in each quadrant was determined using an integrated method. Patients with glaucoma were assigned to three subgroups: initial, asymmetrical and severe glaucoma. Regions of interest (ROIs) were determined anatomically. fMRI (3 T) was performed using a bilaterally presented polar angle stimulus, and the accompanying changes in blood oxygen level-dependent (BOLD) signals were obtained from the occipital poles and calcarine ROIs. We used generalized estimation equation models to compare anatomical and functional data between the groups.

**Results:**

A total of 25 subjects were enrolled, of whom 17 had glaucoma and 8 were controls. Significant associations between quadrant binocular VF sensitivities and fMRI responses were found in the occipital pole ROIs (*p* = 0.033) and the calcarine ROIs (*p* = 0.045). In glaucoma severity subgroup analysis, retinal nerve fiber layer (RNFL) thickness was associated with the BOLD response of the calcarine and occipital pole ROIs (*p* = 0.002 and 0.026, respectively). The initial and asymmetrical glaucoma subgroups had similar binocular VF sensitivities and RNFL thicknesses, but distinct BOLD responses.

**Conclusions:**

The response of the visual cortex to binocular stimulation was associated with binocular VF sensitivity. RNFL thickness was associated with the BOLD response of the calcarine and occipital pole ROIs.

## Introduction

Glaucoma is a major cause of irreversible blindness worldwide and an important cause of visual disability.[1] It is believed that patients with advanced glaucoma suffer from reduced mobility,[2] a higher risk of falling [3], and an increased risk of causing or being involved in automobile accidents. [4] Most patients with glaucoma are unaware of their visual field (VF) defects until the disease enters a late stage.[5,6] As binocularity can compensate for some unilateral VF defects, it is assumed that cortical plasticity allows even the adult brain to compensate for VF defects.[5]

Recent evidence suggests that glaucoma affects the entire visual pathway.[7,8] Studies in primate models of experimental glaucoma [9–13] have demonstrated glaucoma-induced damage in the lateral geniculate nucleus (LGN) and primary visual cortex. Recently, a post-mortem study of a glaucoma patient demonstrated neural degeneration of the optic nerve, LGN and visual cortex, these changes being correlated with clinical and neuroradiological findings.[14]

Improvements in neuroimaging techniques allow new aspects of glaucomatous damage to be studied. High-field magnetic resonance imaging (MRI) enables detailed in vivo evaluation of the central nervous system, allowing detection of subtle variations in brain anatomy and functioning via the performance of functional (f) MRI.[15] An fMRI signal is based on the blood oxygenation level, and is termed BOLD (blood oxygen level-dependent). Such a signal is sensitive to differences in the relative concentrations of oxyhemoglobin (Oxy-Hb) and deoxyhemoglobin (Dx-Hb), both of which have distinct magnetic properties. When the [Dx-Hb]/[Oxy-Hb] ratio falls, the magnetic resonance signal increases. The relationship between neuronal activity and BOLD signal strength is indirect, and is associated with a hemodynamic response induced by synaptic and electrical activity. In simple terms, a BOLD signal reflects the hemodynamic response to the increased metabolic demand from neurons in a specific region of the brain.[16,17]

Along the visual pathway, the final location in which information from either eye is spatially segregated is the LGN. In the primary visual cortex, monocular geniculocortical cells form ocular dominance columns in layer 4C, and next converge into binocular cells in other layers.[18] Previous work using fMRI indicated that cells in the ocular dominance columns may interact if they were simultaneously activated, and that the BOLD response to simultaneous binocular stimulation was lower than that to alternate monocular stimulation.[19] In addition, a substantial BOLD response elicited by stimulation of the opposite eye was observed, possibly associated with spreading of the BOLD signal through the columns, and binocular interactions.[20]

Few studies have used fMRI to study glaucoma. Most delivered monocular visual stimuli and compared the responses of the most affected eye with those of the fellow eye.[21–23]

When studying glaucoma via fMRI, certain aspects of the disease must be considered. First, glaucoma is usually bilateral [24], and the less-affected eye may thus not be the best choice for the control [10]. Second, in real life, both eyes are used together. When the existence of binocular interactions in the visual cortex is considered, it follows that the fMRI response to a binocular visual stimulus may differ from that to a monocular stimulation. Hence, it is possible that the fMRI response to binocular visual stimulation is distinctive in patients with glaucoma.

The aim of the present study was to assess the fMRI response to visual stimuli presented bilaterally to glaucoma patients and controls, and the associations thereof with structural ocular findings and psychophysical test data.

## Methods

This was a cross-sectional study. Written informed consent was obtained from all of the subjects. The research followed all of the tenets of the Declaration of Helsinki and was approved by the Ethics Committee of the Israeli Albert Einstein Hospital and the Federal University of São Paulo, São Paulo, Brazil.

Ocular examinations included measurement of best-corrected visual acuity (BCVA), dilated stereoscopic fundoscopy, intraocular pressure (IOP) measurement via Goldmann applanation tonometry, standard automatic perimetry (SAP) using the Humphrey Swedish Interactive Threshold Algorithm (SITA) strategy 24–2 test, and optical coherence tomography (OCT, Zeiss-Humphrey Instruments, Dublin, CA).

Inclusion criteria for the glaucoma group were: age >18 years; BCVA of 20/40 (0.3 logMAR) or better; a spherical equivalent ±6 D; and the presence of primary open angle glaucoma (POAG) defined as follows:

- elevated IOP (>21 mmHg) measured on two occasions when the patient was not on medication; and
- a glaucoma hemifield test (GHT) result outside of normal limits; or a pattern standard deviation (PSD) with a value of *p*<0.05; or a cluster of three points or more in the PSD plot in a single hemifield, with a value of *p*<0.05, one of which had a value of *p*<0.01 in one or both eyes; and
- a cup/disc ratio greater than 0.6 and/or a nerve fiber layer defect with a corresponding optic disc change, in one or both eyes.

Exclusion criteria were: no reliable VF; previous corneal surgery, complicated cataract surgery, or any surgery performed less than 3 months from the beginning of the study; presence of retinal disease; use of an alpha-adrenergic agent; a neurological or psychiatric disease; drug dependence; use of controlled medication; tremor or dystonia that prevented performance of MRI; claustrophobia; implantation of a metallic device, including a pacemaker, aneurysm clip, or a cochlear implant; presence of lesions in the brain parenchyma that were evident at the commencement of the study, except for discrete punctiform dots in the white substance or a slight reduction in cortical volume.

The control group consisted of healthy subjects of similar age, gender ratio, and schooling level as the test group; with IOPs <22 mmHg; normal visual fields; cup/disc ratios <0.6; differences in between-eye cup/disc ratios of <0.2; BCVA of 20/40 (0.3 logMAR) or better; and spherical equivalents ±6 D.

The exclusion criteria were identical to those applied to the glaucoma group.

Glaucoma severity was classified using the scheme of Hodapp, Parrish, and Anderson [25]. To improve the analysis of the glaucoma group, the patients were assigned to three subgroups: (1) initial glaucoma, (2) asymmetrical glaucoma and (3) severe glaucoma, according to the VF-defect pattern in both eyes.

### fMRI evaluation

**Fig 1:**
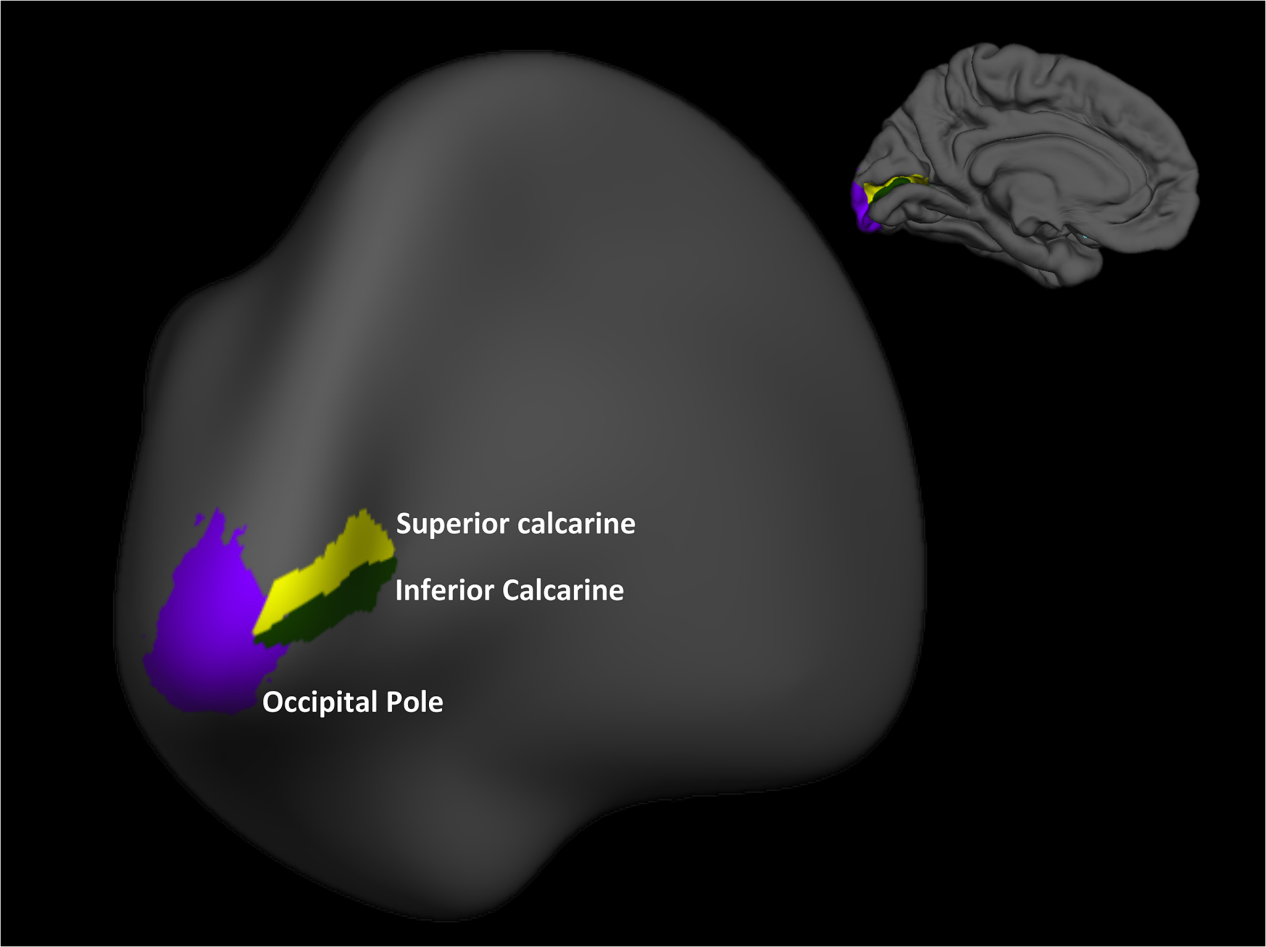
Freesurfer software segmentation of the visual cortex and regions of interest investigated: the occipital pole and the superior and inferior calcarine regions.

All MRI was performed using a 3.0-Tesla instrument (Siemens TIM Trio) with a gradient amplitude of 45 mT/m and a slew ratio of 150 mT/m/s. A reception head coil containing 12 elements was used for image acquisition. Both anatomical and functional scans were obtained. Volumetric T1-weighting images (MPRAGE, TR/TE/TI/FA/VOXEL = 2,500 ms/3.45 ms/1,100 ms/7°/1×1×1 mm^3^) were automatically processed using the Freesurfer image analysis suite, version 5.1.0, which is well-documented and freely available online (http://surfer.nmr.mgh.harvard.edu/). ROIs were determined anatomically via automatic segmentation using the Aparc.a2009s atlas [26] and used in the fMRI analysis. The ROIs used were the occipital pole and the superior and inferior calcarine regions of each hemisphere (Fig 1).

**Fig 2:**
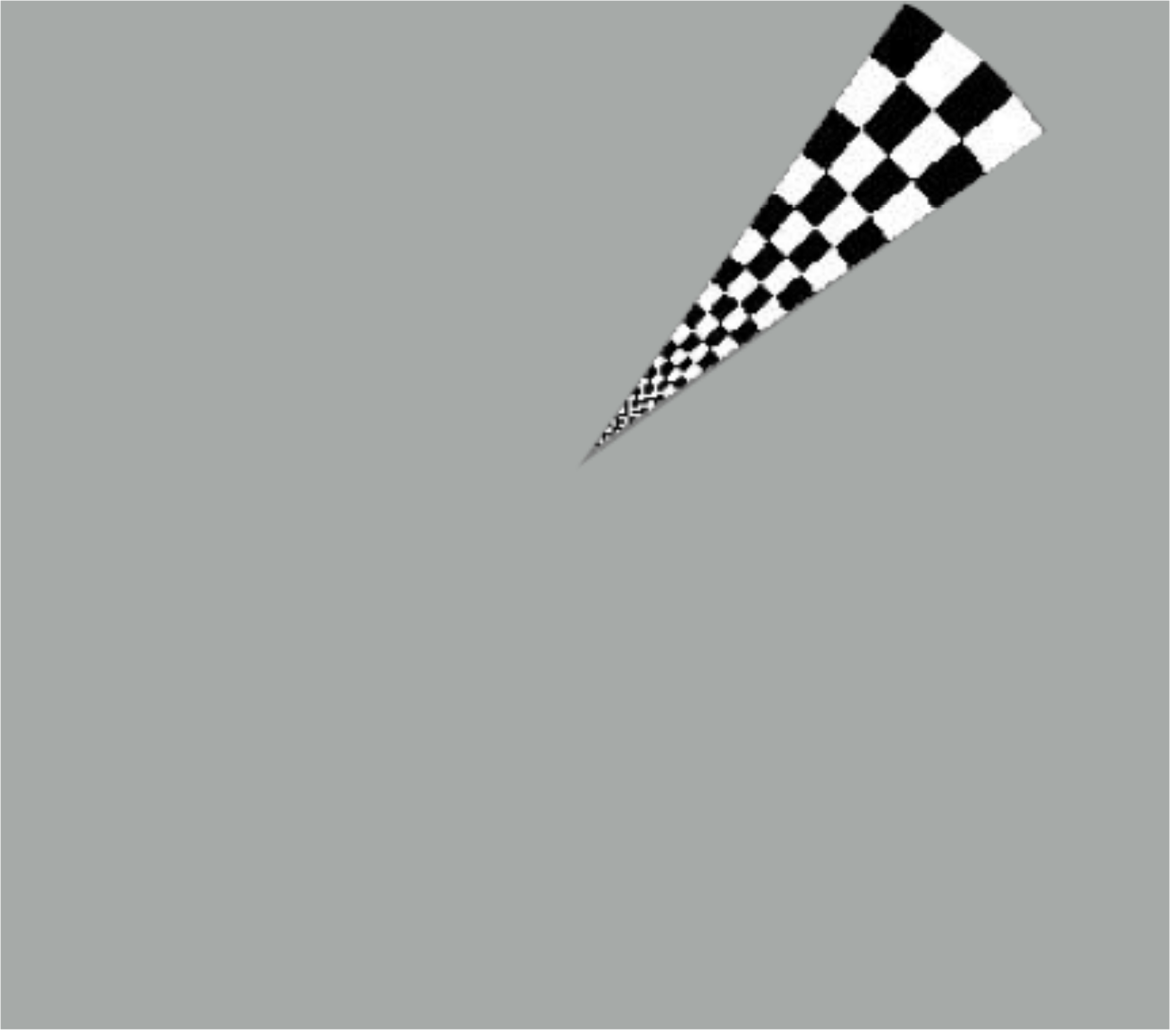
Polar angle visual stimuli used during functional magnetic resonance imaging scanning.

We obtained T2*-weighted BOLD images (GE-EPI, TR/TE/FA/GAP/VOXEL = 2,000 ms/30 ms/83°/0.5 mm/3×3×3 mm^3^) via fMRI. A binocular system was used to present visual stimuli. The scan frequency was 85 Hz, at 2.3 min of arc, and the total visual field extended 30° horizontally and 23° vertically (http://www.nordicneurolab.com; NNL, Norway). The fMRI paradigm was a polar angle stimulus consisting of a rotating wedge, featuring a contrast-reversing checkerboard pattern of 8 Hz, and 100% contrast, rotating clockwise around a fixation target (Fig 2). This was superimposed on a homogeneous gray background of the mean intensity of the checkerboard stimulus. The stimulus was presented bilaterally over three cycles of 60 s each, and, during the rest phase, each individual was instructed to fixate on a small central dot on the gray background screen. The system was controlled and synchronized by an opto-electrical trigger controlled by the RF signal from the MR system (Zurc&Zurc, São Paulo, Brazil) running in-house software. During stimulus presentation, the subjects were instructed to fixate on a target at the center of the screen and to press a button whenever the target changed (from a cross to a point and vice-versa), to maintain fixation; this ensured that the participants focused on the centers of their VFs. Each visual stimulus activated a specific area of the occipital cortex, creating traveling waves of neural activity in the visual cortex. For example, when a stimulus was presented to the superior right quadrant, the left occipital pole and left inferior calcarine region were activated. All of the subjects were closely monitored during fMRI examination.

FMRI data processing was performed using FEAT (FMRI Expert Analysis Tool) Version 5.98, part of the FSL (FMRIB's Software Library, www.fmrib.ox.ac.uk/, version 4.0.1). Pre-processing steps included correction of head movement, spatial smoothing using a Gaussian kernel of FWHM 6.0 mm, and high-pass temporal filtering. Statistical analysis of whole-brain data was performed using a GLM (General Linear Method) implemented in the FSL package. Four exploratory variables were used, one for each quadrant. Next, we used the FEATQUERY tool to extract BOLD data for each ROI.

The interval between ocular examination and fMRI never exceeded 6 months.

### Visual field testing

Standard automated perimetry (SAP) was performed using the Humphrey field analyzer and the 24–2 SITA method. All participants had undergone at least two prior VF SAP tests. Subjects with fixation losses >20%, or a >33% rate of false-positive or-negative responses, were excluded from the study.

Binocular VF can be estimated using monocular tests. The best location and binocular summation methods enable reliable predictions of the binocular VF.[27–29] In the present study, the binocular VF was measured using the best location (or integrated) VF method. Thus, at each location, the highest between-eye VF sensitivity was recorded.

Differences (in decibels) at each point in the total deviation binocular field were converted, point-by-point, to a linear scale, using logarithmic transformation. Averaged per-quadrant VF sensitivities (on a linear scale) were calculated.

### Retinal nerve fiber layer (RNFL) thickness

RNFL thickness was determined via optical coherence tomography (OCT3, Stratus, Carl Zeiss Meditec, Inc.), using the fast RNFL circular scan mode. The mean superior (45° to 135°) and inferior (225° to 315°) RNFL thicknesses were used in the analysis and compared to the BOLD signals.

### Statistical analysis

To compare the anatomical and functional data between the control and glaucoma groups (with and without severity subgroup analysis), generalized estimating equation (GEE) models [30,31] were used to correct for dependencies between the two eyes, among the four quadrants of each eye (when psychophysical tests were performed), between the two hemi-retinas of each eye (on OCT analysis) and among the four segments of the occipital cortex. Because the GEE does not provide a correlation coefficient, beta values were calculated to determine which independent variable had a greater effect on the dependent variable. Quadrant binocular VF and RNFL thickness were the independent variables. The BOLD response was the dependent variable. A value of *p*<0.05 was taken to indicate statistical significance.

## Results

Twenty-five individuals were enrolled in the study; these included 17 glaucoma patients and 8 healthy controls. Baseline characteristics of the groups are shown in Table 1. Classification of VF defects according to Hodapp, Parrish and Anderson [25] and subgroup divisions are shown in Table 2.

**Table 1:** Baseline characteristics of control group and glaucoma group.

	Control Group	Glaucoma Group	*p* value
**N**	8	17	
**Male/Female**	5/3	7/10	0.319
**Age, mean ± SD**	56.4 ± 13.9	61.8 ± 10.9	0.301
**BCVA (logMAR)**			
right eye	0.05 ± 0.08	0.08 ± 0.10	0.355
left eye	0.04 ± 0.05	0.1 ± 0.1	0.347
**Average RNFL thickness**			
right eye	96.28 ± 14.63	72.11 ± 20.41	0.397
left eye	96.27 ± 15.84	72.09 ± 15.15	0.705
**SAP MD, mean ± SD**			
right eye	-1.75 ± 0.89	-9.69 ± 9.92	0.003
left eye	-1.11 ± 0.93	-9.21 ± 6.80	0.012
**Race**			0.838
caucasian	6	10	
asian	1	4	
black	1	3	

BCVA = best corrected visual acuity; logMAR = logarithm of the minimal angle of resolution; SD = standard deviation; RNFL = retinal nerve fiber layer; SAP = standard automated perimetry; MD = mean deviation; dB = decibel.

**Table 2:** The severities of visual field defects in both eyes of the glaucoma group and subgroup classification.

Better eye	Poorer eye	Subgroup classification	N
Normal/Early	Moderate	Initial glaucoma	3
Normal/Early	Severe	Asymmetrical glaucoma	8
Moderate/Severe	Severe	Severe glaucoma	6

**Fig 3:**
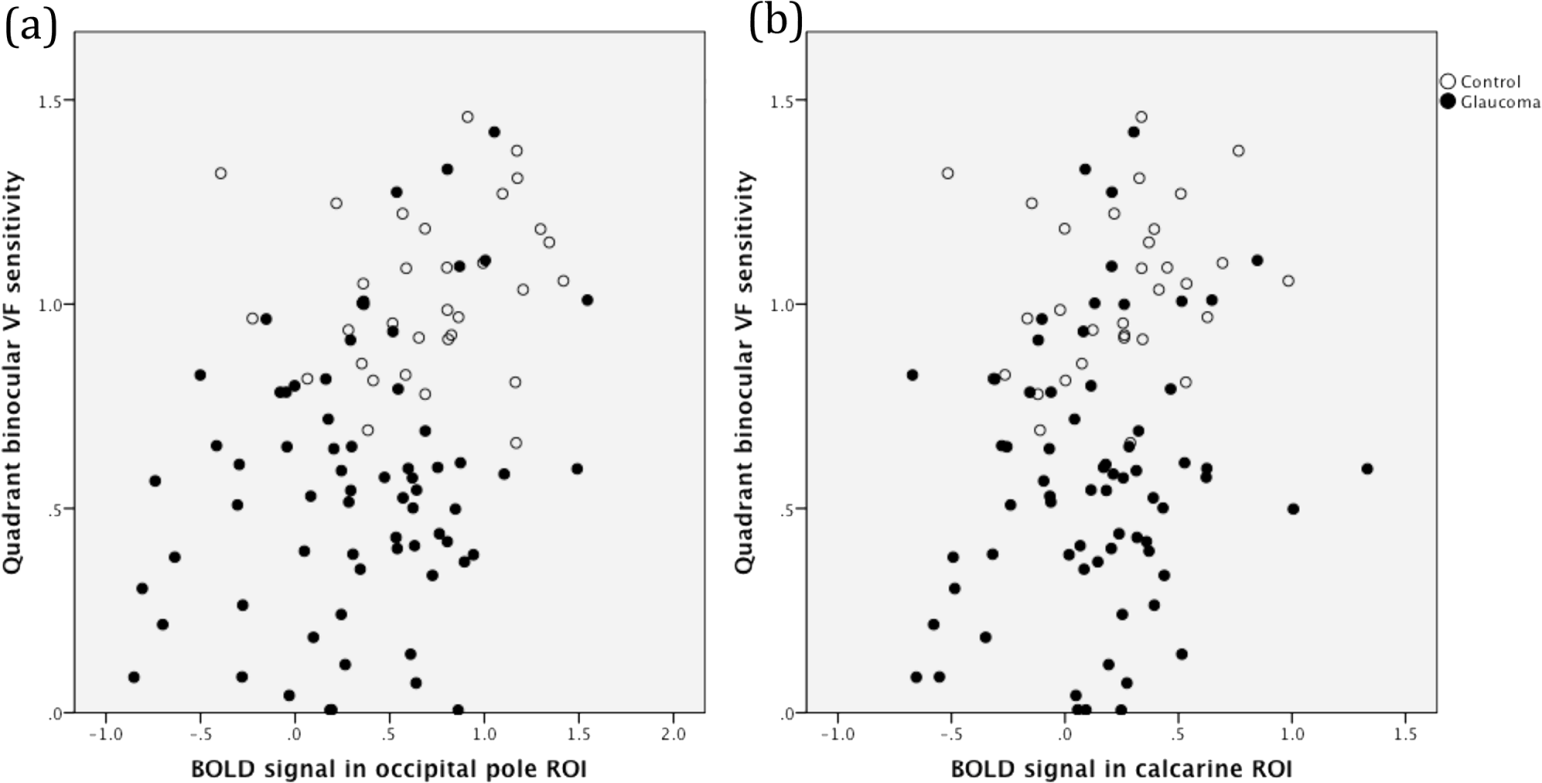
Quadrant binocular visual field (VF) sensitivities and blood oxygen level-dependent (BOLD) signals from the regions of interest (ROIs) of the glaucoma and control groups: (a) occipital pole ROIs; (b) calcarine ROIs.

The response (the BOLD signal) of the occipital cortex to a visual stimulus was evaluated in the two ROIs, with reference to the position of a polar angle stimulus applied for a given time. A statistically significant association was evident between binocular VF sensitivities and BOLD signal strengths from the two ROIs (Table 3), but no between-group difference was noted (*p* = 0.900 for the calcarine ROI and *p* = 0.203 for the occipital pole ROI). The results are summarized in Table 3 and Fig 3.

**Table 3:** Associations (*p* values and betas) between BOLD signals from the regions of interest (ROIs), and the independent variables (quadrant binocular VF sensitivities and study group), calculated using generalized estimating equation models.

ROI	Independent variable	Effect on BOLD (Beta ± SE)	*p* value
**Occipital Pole**	quadrant binocular VF	0.409 ± 0.19	0.033
** **	group (glaucoma/control)	0.188 ± 0.15	0.203
**Calcarine**	quadrant binocular VF	0.235 ± 0.12	0.045
	group (glaucoma/control)	-0.013 ± 0.10	0.9

ROI = region of interest; SE = standard error; VF = visual field.

**Fig 4:**
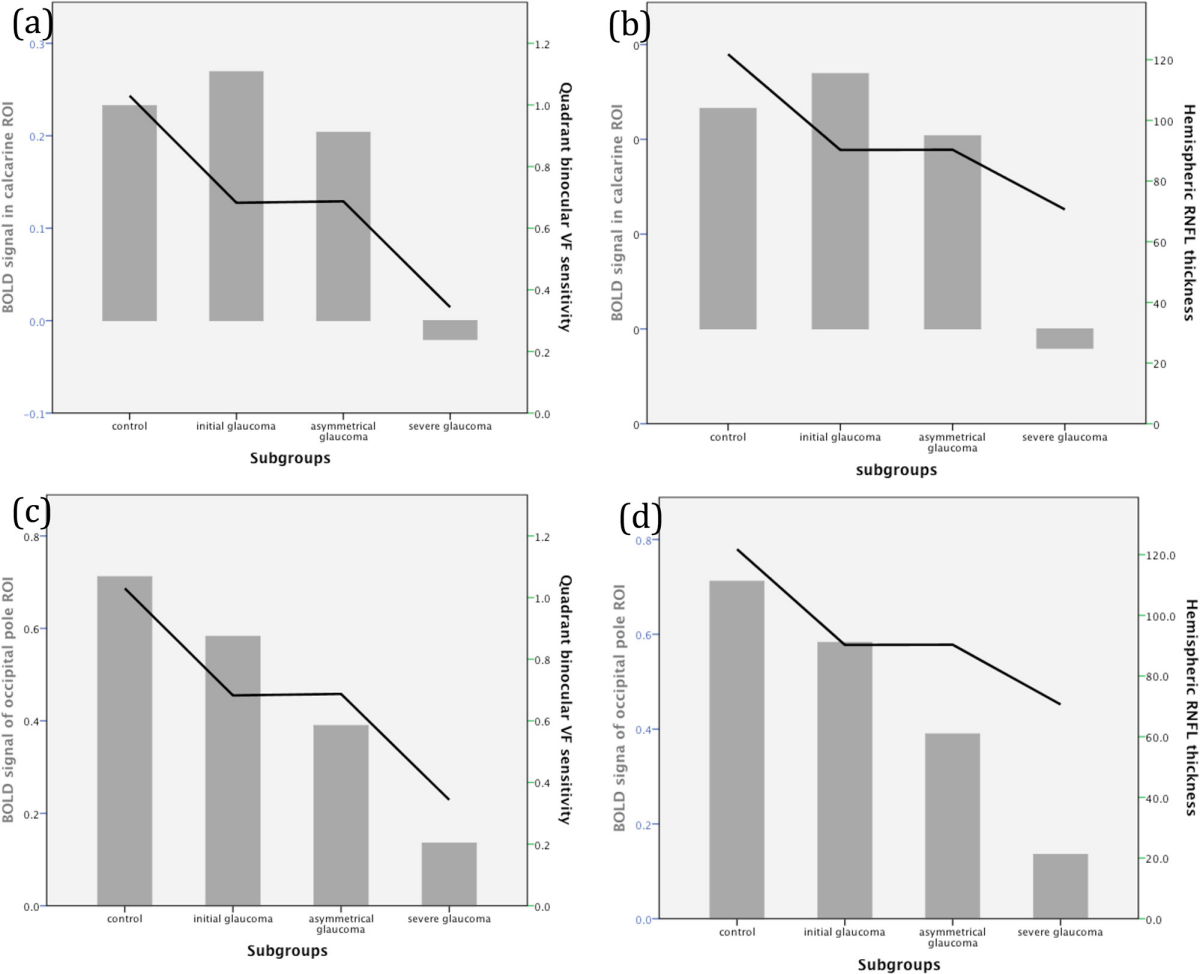
Subgroup analysis demonstrating: (a) Blood oxygen level-dependent (BOLD) signal in calcarine regions of interest (ROIs) and binocular visual field (VF) sensitivity; (b) BOLD signal in calcarine ROIs and hemispheric RNFL thickness; (c) BOLD signal in occipital pole ROIs and binocular VF sensitivity; and (d) BOLD signal in occipital pole ROIs and hemispheric RNFL thickness.

When the glaucoma group was subdivided, the BOLD signal of the occipital pole ROIs of the severe glaucoma group was statistically significantly different compared to the control (*p*<0.001) and initial glaucoma groups (*p* = 0.026). The BOLD signal of the calcarine ROIs of the severe glaucoma group was almost significantly different compared to the control group (*p* = 0.059) and was significantly different compared to the initial glaucoma group (*p* = 0.033). The apparent increase in BOLD signal strength of the calcarine ROI from control group to initial glaucoma group had no statistical significance (p = 0.758). The distributions of the BOLD responses of the subgroups are shown in Fig 4.

In addition, the binocular VF sensitivity and mean hemispheric RNFL thickness were similar in the initial and asymmetrical glaucoma groups, whereas the BOLD signal strength was lower in the asymmetrical glaucoma group (Fig 4).

The mean hemispheric RNFL thicknesses of both eyes, determined by OCT, were associated with the BOLD responses of the calcarine and occipital pole ROIs (*p* = 0.015 and 0.026, respectively), in subgroup analysis.

## Discussion

This study addressed the visual cortex response (BOLD signal) to binocular VF stimulation. We found a significant association between BOLD signal strength and binocular VF sensitivity in the ROIs studied.

Few fMRI studies have been conducted on glaucoma patients.[21–23,32–34] The first such work, comparing VF defects with fMRI responses, included seven patients with various forms of optic neuropathy, including glaucoma. It was found that stimulation of the affected eye did not activate the primary visual cortex in the region corresponding to the VF defect [34]. Duncan et al. [23] studied six cases of asymmetric POAG using 3-Tesla fMRI. V1 landmarks were first identified using standard binocular stimuli, and templates were fitted for the patients. The borders of the more damaged quadrants of the VFs of glaucomatous eyes were projected onto flattened cortical surfaces, and defined as ROIs. Scotoma-mapping stimuli were presented alternately to either eye and the resulting BOLD signals from the ROIs were compared to interocular PSD differences. In addition, a pointwise analysis of 12 pattern deviation values and the BOLD amplitude was performed, comparing the most affected quadrant of the glaucomatous eye with the same quadrant of the fellow eye. The cited authors found that the BOLD signal from the human V1 was altered in a manner consistent with loss of the VF. The amplitude of the BOLD response correlated pointwise with the differences in sensitivity thresholds between the glaucomatous and fellow eye. In contrast to our approach, in which ROIs were determined anatomically, the cited studies used functional localizers to delineate V1 landmarks, and the ROI was defined as the projection of the most affected VF quadrant of the glaucomatous eye (the same quadrant of the fellow eye served as a control).

Qing et al. [22] investigated fMRI responses in the primary visual cortices of six POAG patients with asymmetric VF damage and spared central vision, using the fellow eyes as controls. The BOLD signals from the affected eyes were lower in the test group, and negatively correlated with the interocular PSD difference.

The cited studies used methods that differed in some aspects from those in the present study. The ROI determination was performed anatomically in our study, not functionally. A close agreement between anatomically and functionally derived V1 boundaries was described, indicating that V1 can be accurately predicted based on the cortical surface reconstruction computed from structural MRI scans[35]. Several patterns of VF stimulation were used in fMRI studies. The visual stimulus used in the present study was the polar angle, because it enables the evaluation of the VF quadrants and the comparison with the correspondent region in the visual cortex.

Some studies reported an asymmetry of the BOLD response in the VF, with a stronger response in the lower than the upper VF [36,37]. In the present study, this was unimportant, because we compared the sensitivity of the VF with the BOLD response in the anatomically correspondent region, and not the superior with the inferior VF.

Negative and positive BOLD signals were observed in V1 because of a distinct neurovascular coupling mechanism [38]. In the present study, we used anatomical landmarks to analyze the BOLD signal in V1. This approach allows for direct anatomical and functional correlations and, because of between-subject differences in the cytoarchitecture, areas with a negative BOLD response may be included in the ROIs.

Even when considering the differences in methodologies, our data are consistent with earlier findings of a reduction in the cortical response when the VF sensitivity diminishes. The association between binocular VF and the BOLD response was statistically significant. Although no significant difference in the BOLD signal strength between the control and glaucoma groups was found, subgroup analysis elucidated the pattern of the BOLD response in the different categories of glaucomatous damage. The initial and asymmetrical glaucoma groups had similar binocular VF sensitivities and mean hemispheric RNFL thicknesses, but distinct BOLD responses, with a lower BOLD signal strength in individuals with asymmetrical glaucoma. The decreased BOLD response may imply reduced neural activity of the cells that receive afferent input from the most affected eye in the asymmetrical glaucoma group.

An association was evident between RNFL thickness and the BOLD signal of the calcarine and occipital pole ROIs in subgroup analysis. Any association between RNFL thickness and the cortical functional response remains controversial. Duncan et al. found significant associations between inter-eye differences in OCT RNFL thickness, Heidelberg retinal tomographic data, retinal polarimetric data, and the BOLD amplitude [21]. In contrast, Qing et al. found no association between the BOLD signal and RNFL thickness as measured by OCT, GDX-VCC, or HRT-II[22]. Each study included six POAG patients with asymmetric visual fields.

A limitation of the present study was the heterogeneity of the glaucoma group. The disease severity varied widely in both eyes, which may have been responsible for the lack of a significant difference in BOLD signal and average RNFL thickness between the glaucoma and control groups. A further limitation was the small sample size. Although we had one of the larger sample sizes compared to the published studies using fMRI to investigate glaucoma, our sample size was still small, particularly for subgroup analysis. In addition, it would be interesting to deliver both bilateral and unilateral visual stimuli to elicit BOLD responses, although this would make the MRI scan time too long.

In conclusion, we provided evidence that the occipital cortical response to binocular stimulation is positively associated with binocular VF sensitivity and hemispheric RNFL thickness. Studies using larger sample sizes are required to confirm the altered visual cortex activity in different degrees of glaucoma.
